# Diffuse Low-Grade Glioma With Spontaneous Radiological Regression: A Case to Be Followed

**DOI:** 10.7759/cureus.96671

**Published:** 2025-11-12

**Authors:** Abdelkouddouss Laaidi, Oufaa Jamal, Abd El Hamid Jehri, Marouane Makhchoune, Mehdi Karkouri, Khadija Ibahioin, Said Hilmani, Abdessamad Naja, Abdelhakim Lakhdar

**Affiliations:** 1 Neurosurgery, University Hospital of Casablanca, Casablanca, MAR; 2 Neurosurgery, Ibn Rochd University Hospital Center, Casablanca, MAR; 3 Anatomopathology, University Hospital of Casablanca, Casablanca, MAR

**Keywords:** cannabinoids and gliomas, diffuse low-grade glioma, glioma prognosis, spontaneous regression, steriotactic biopsy

## Abstract

Low-grade gliomas (LGGs) are slow-growing, infiltrative brain tumors with a tendency to progress over time. Spontaneous regression of LGG is an exceptionally rare phenomenon, with only one case documented in the literature. The mechanisms underlying this phenomenon remain unclear but may involve immunological responses, spontaneous apoptosis, vascular remodeling, or hormonal influences, and may open up some new therapeutic perspectives for the future. We report the case of a 21-year-old woman with no medical history who presented with headaches, visual disturbances, and progressive left-sided hemiparesis. Brain MRI revealed a diffuse glioma in the splenium of the corpus callosum, extending into the parieto-rolandic region. A stereotactic biopsy confirmed the diagnosis of a diffuse LGG (not otherwise specified (NOS)). The patient received only symptomatic treatment with anticonvulsants and five days of corticosteroids, without adjuvant therapy. At the six-month follow-up, she showed significant neurological improvement, and imaging demonstrated partial tumor regression. By the one-year follow-up, the lesion had nearly disappeared, with no signs of recurrence. A histopathological review confirmed the initial diagnosis, ruling out other possible differential diagnoses. Spontaneous regression of diffuse LGG remains an extremely rare event with unclear underlying mechanisms. This case underscores the complexity of glioma biology and highlights the need for further research to explore potential predictive biomarkers and alternative therapeutic strategies based on the pathways involved in spontaneous regression.

## Introduction

Low-grade gliomas (LGGs) are a heterogeneous group of infiltrative primary brain tumors that arise from glial cells and account for approximately 15-20% of all gliomas. According to the 2021 World Health Organization (WHO) classification, low-grade diffuse gliomas are defined through an integrated diagnosis that combines histopathological features with key molecular alterations. These tumors primarily include isocitrate dehydrogenase (IDH)-mutant astrocytomas and oligodendrogliomas with combined IDH mutation and 1p/19q co-deletion, which correspond to WHO grade 2 lesions within the spectrum of adult-type diffuse gliomas [1,2].

Clinically, LGGs typically affect young adults and often present with seizures or progressive focal deficits. Despite their indolent nature, they are considered “progressive, invasive, and chronic” diseases of the central nervous system (CNS) that inevitably undergo malignant transformation over time through cumulative genetic and epigenetic alterations [3,4]. Current management strategies emphasize maximal safe resection, followed by individualized decisions regarding adjuvant radiotherapy or chemotherapy, based on residual tumor volume, molecular profile, and patient factors.

Spontaneous regression of intracranial gliomas is exceptionally rare, and most documented cases involve NF1-associated optic pathway, thalamic, or pediatric brainstem gliomas [3-5]. Only one case of spontaneous radiological regression in an adult with a supratentorial diffuse low-grade glioma (DLGG) has been reported [4], underscoring the exceptional nature of this phenomenon.

In this article, we describe a unique case of a supratentorial diffuse low-grade glioma (DLGG) in an adult patient that exhibited radiological regression without any adjuvant therapy. This presents a compelling opportunity to explore spontaneous glioma regression beyond the conventional contexts recognized in NF1 or pediatric pontine tumors. We also conducted a systematic review of the literature to contextualize our findings and examine proposed mechanisms, such as biopsy-induced regression, immunomodulatory effects, hormonal influences, and other biological factors.

## Case presentation

A 21-year-old woman, with no relevant medical history except for a cannabis-smoking brother, presented with a two-month history of headaches and decreased visual acuity. Approximately three weeks after the onset of headaches, she noticed a progressive weakness in her left arm and leg. This was followed by two episodes of seizures, which were successfully managed with anticonvulsant therapy, the week preceding her hospitalization.

**Figure 1:**
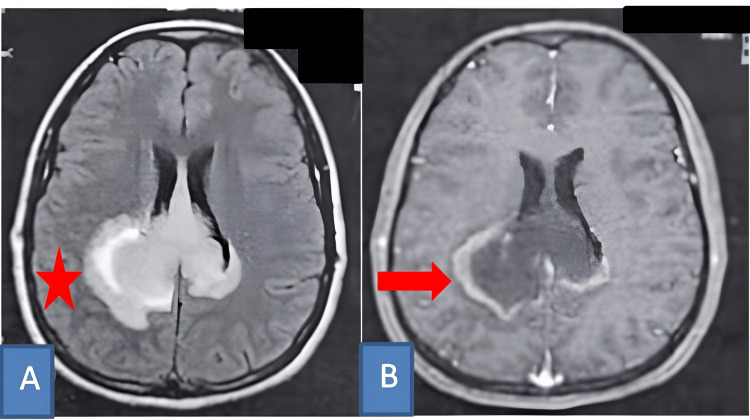
Magnetic resonance imaging (MRI) of the brain showing a neoplastic lesion centered in the splenium of the corpus callosum with parietal lobe extension. (A) Axial FLAIR sequence demonstrating a hyperintense lesion (red star). (B) Axial T1-weighted sequence after gadolinium administration showing a hypointense lesion with peripheral contrast enhancement (red arrow). The imaging features are consistent with a low-grade glioma with partial enhancement and extension toward the parietal white matter. FLAIR: fluid-attenuated inversion recovery.

Neurological examination revealed a conscious patient with hemiparesis on the left side, graded at 3/5, with no other significant findings. Brain MRI demonstrated a lesion suggestive of a neoplastic process located in the splenium of the corpus callosum, with infiltration into the parieto-rolandic region. On axial fluid-attenuated inversion recovery (FLAIR) sequences, the lesion measured 59 mm x 34 mm. It was characterized by T1-weighted hypointensity, T2/FLAIR hyperintensity, and exhibited peripheral contrast enhancement following gadolinium administration (Figure 1).

**Figure 2:**
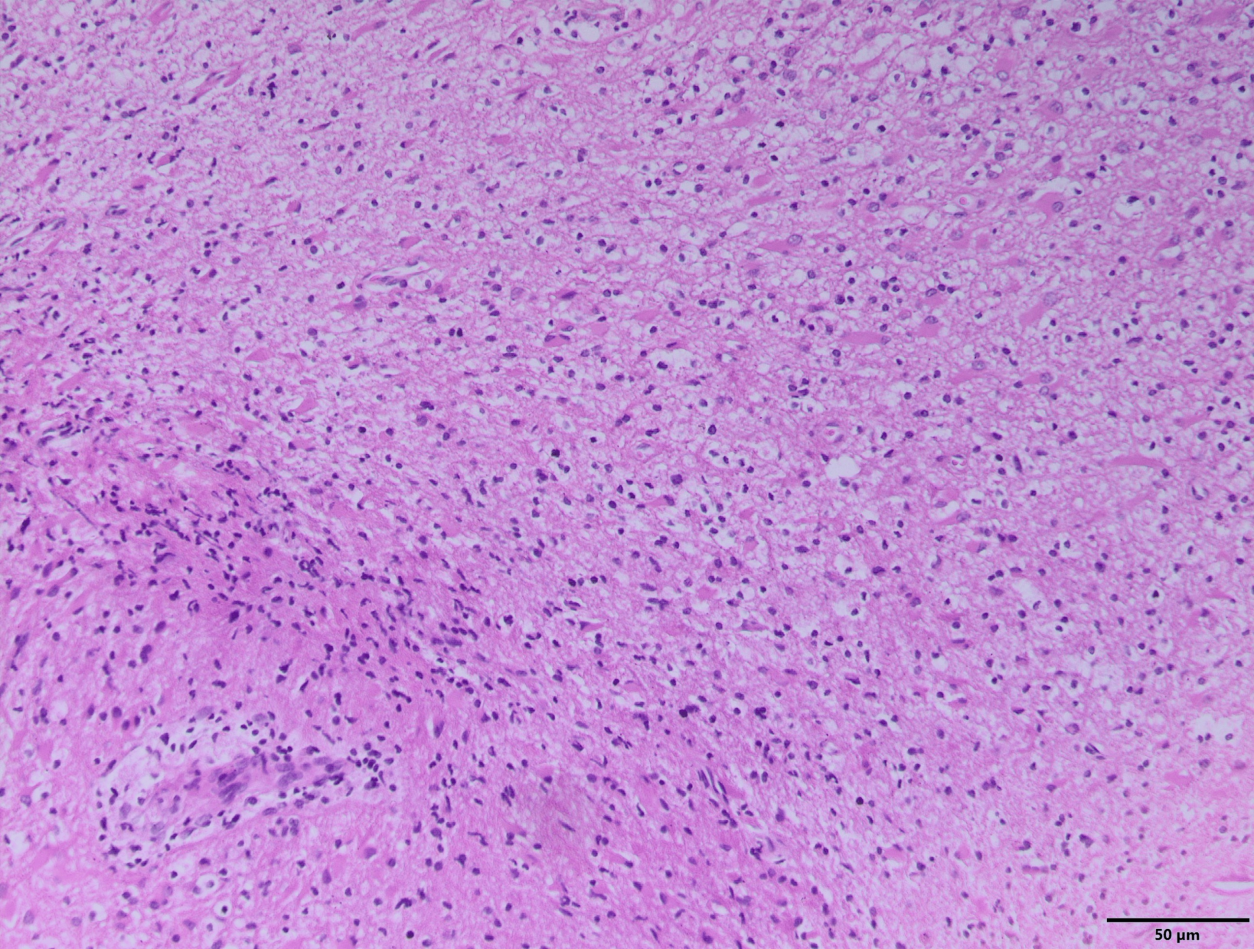
Representative photomicrograph of the tumor showing features consistent with a low-grade glioma (hematoxylin and eosin stain, ×200 magnification). The section reveals moderate cellularity composed of uniform glial cells with elongated nuclei and fibrillary cytoplasmic processes, embedded in a fibrillary stroma. No necrosis, microvascular proliferation, or significant mitotic activity is observed, supporting the diagnosis of a low-grade (WHO grade II) glioma. The histological architecture shows mild nuclear atypia and the absence of high-grade features.

The patient underwent a stereotactic biopsy. Histopathological examination revealed a fragmented glial biopsy infiltrated by a moderately cellular glial tumor set within a fibrillary stroma. Neoplastic cells exhibit oligodendroglial morphology with characteristic clear pericellular halos and hyperchromatic nuclei. Numerous gemistocytic cells are present, alongside only mild atypia. Vascular structures are thin, with no endothelial proliferation. No necrosis or mitotic activity is observed. A focal perivascular lymphocytic infiltrate is noted (Figure 2).

Immunohistochemical analysis confirmed the glial origin of the lesion, with diffuse positivity for glial fibrillary acidic protein (GFAP) and OLIG2. Nuclear expression of ATRX was retained, while both p53 and IDH1 (R132H) immunostaining were negative. The Ki-67 proliferation index was low, estimated at <1%. Unfortunately, molecular testing for 1p/19q co-deletion could not be performed at the time of diagnosis due to technical limitations.

Taken together, these histological and immunophenotypic findings support the diagnosis of a diffuse low-grade glioma, not otherwise specified (NOS), according to the 2021 World Health Organization classification of central nervous system tumors, while effectively excluding alternative entities such as demyelinating lesions or primary CNS lymphoma.

A multidisciplinary neuro-oncology board recommended a strategy of vigilant observation without immediate adjuvant therapy. The patient was placed on corticosteroid therapy for five days and prescribed lamotrigine for seizure management. This conservative approach was predicated on the patient's stable neurological status after initial symptomatic management, the absence of mass effect, and a low proliferative index (<1%).

**Figure 3:**
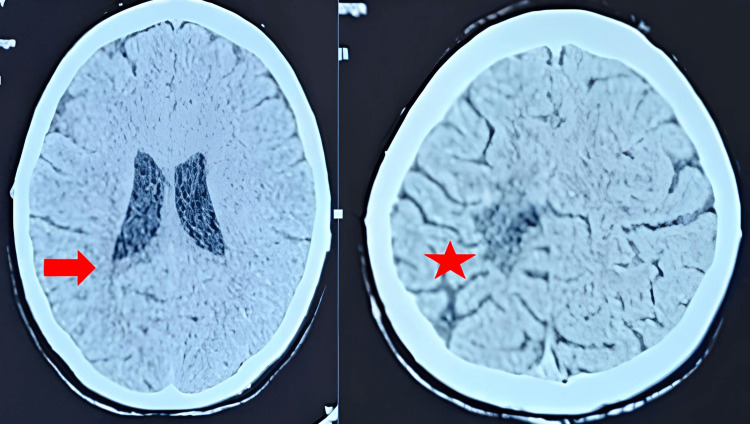
Six-month post-contrast brain CT scan showing partial radiological regression. (A) The red arrow indicates the residual hypodense area in the left parietal region, corresponding to a zone of decreased cellular activity. (B) The red asterisk highlights a smaller enhancing component compared to baseline imaging, consistent with a partial reduction in lesion size and enhancement intensity. These findings reflect ongoing radiological improvement and support the decision to maintain clinical observation rather than initiate adjuvant therapy.

Clinical evaluations were performed monthly, with imaging follow-up scheduled at three and six months. During this period, the patient remained clinically stable, with progressive improvement of motor strength and no new neurological symptoms. The six-month CT scan (Figure 3) confirmed partial radiological regression, supporting the decision to maintain observation rather than initiate adjuvant therapy.

**Figure 4:**
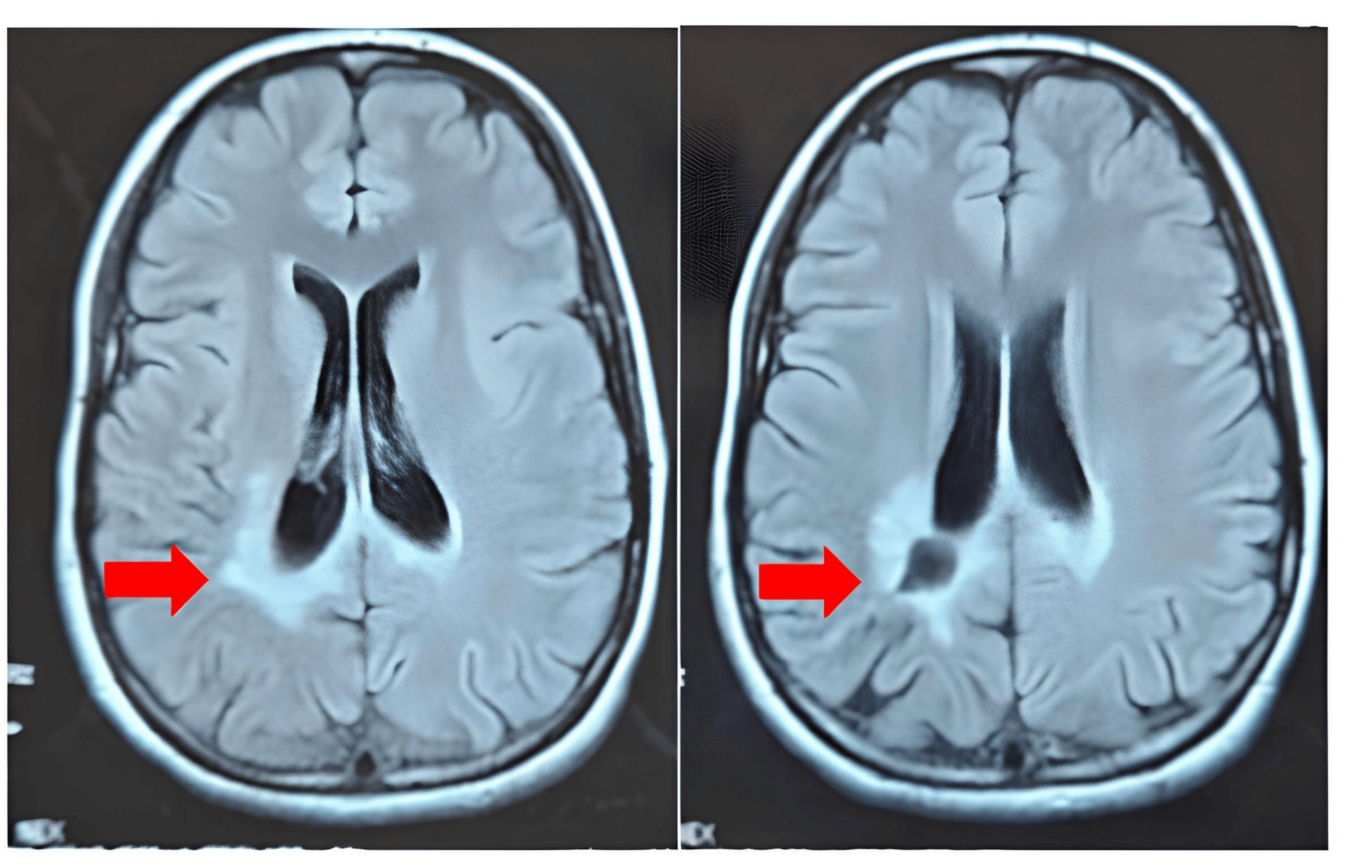
Axial brain MRI FLAIR sequences obtained at 18 months follow-up. Axial brain MRI FLAIR sequences obtained at 18 months follow-up showing near-complete regression of the previously identified lesion in the left parietal region (red arrows). The hyperintense signal seen in earlier scans has markedly decreased, with only minimal residual gliotic changes remaining. FLAIR: fluid-attenuated inversion recovery.

This enabled a reduction in the dosage of anticonvulsant therapy. By 18 months follow-up, the patient exhibited only mild residual crural monoparesis (graded 4+/5) and remained seizure-free without any ongoing anticonvulsant treatment. Follow-up brain MRI revealed near-complete regression of the lesion (Figure 4).

**Figure 5:**
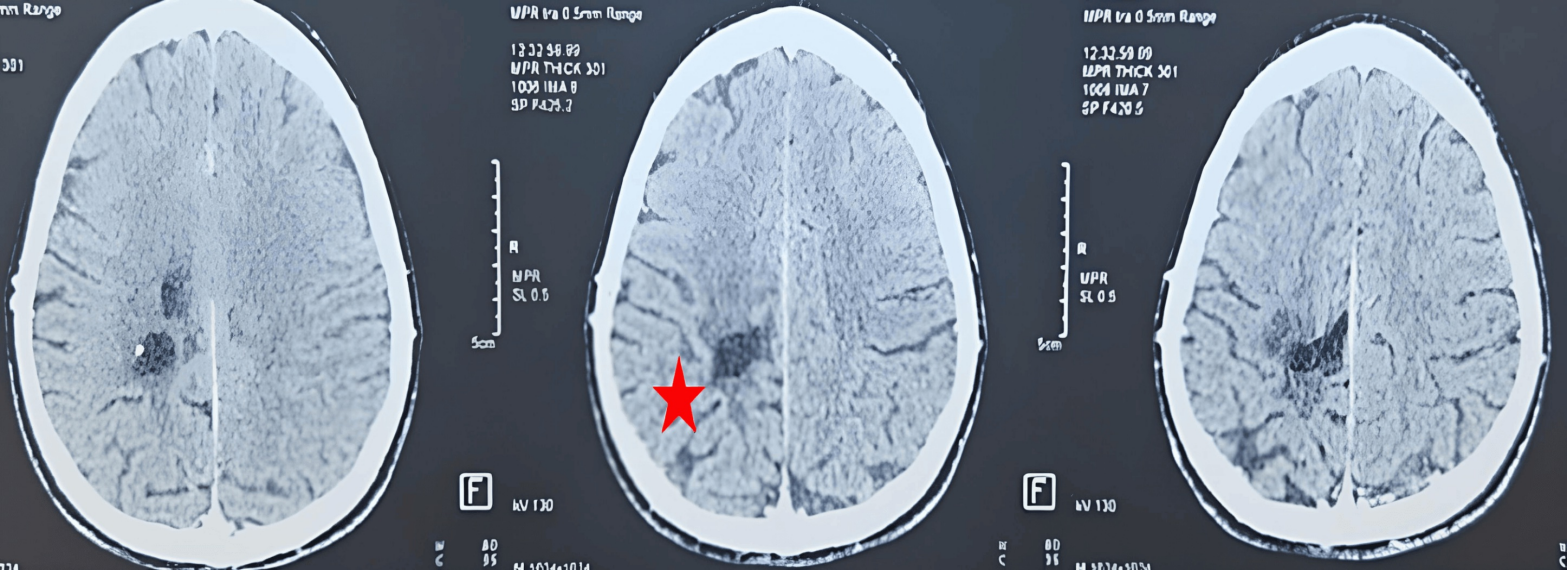
Two-year follow-up brain CT scan. Axial images demonstrate a stable hypodense lesion in the left frontal lobe (red star), without evidence of progression or new contrast enhancement. The overall radiological aspect remains unchanged compared with previous examinations, confirming the long-term stability of the lesion.

Given this unusual clinical presentation and the rarity of spontaneous regression, a biological workup was performed to investigate a potential inflammatory etiology, but the results were normal. A histopathological review was independently performed by two experienced neuropathologists to confirm diagnostic accuracy. Both evaluated the slides for key histological features (cellularity, nuclear atypia, mitotic activity, necrosis, and vascular proliferation) and reviewed immunostaining results, including GFAP, ATRX, p53, and Ki-67 expression. These assessments confirmed the diagnosis of diffuse low-grade glioma and excluded other differential diagnoses, particularly primary central nervous system lymphoma. The two-year medical follow-up shows that the patient is stable both clinically and radiologically (Figure 5).

## Discussion

The spontaneous regression of low-grade gliomas (LGGs) represents an exceptionally rare phenomenon in neuro-oncology. Most previously reported cases have involved pilocytic astrocytomas (WHO grade 1), particularly in association with neurofibromatosis type 1 NF1, or in tumors located within the optic pathways, thalamus, or pediatric brainstem [3,5-8].

Although these circumscribed gliomas differ biologically from diffuse WHO grade 2 gliomas, they remain relevant for discussing potential mechanisms of spontaneous regression, such as immune-mediated responses, apoptosis, hormonal influence, or vascular changes that may also play a role in diffuse gliomas.

​​​​The association with NF1 represents the most frequently observed correlation, with over 15 cases of spontaneous regression documented in NF1-associated LGGs. This genetic disorder stems from either autosomal dominant inheritance or sporadic mutation affecting the tumor suppressor gene at chromosome 17q11.2 [9]. In contrast, for patients without NF1 who develop LGGs, no reliable predictors of regression have been identified.

Also, a systematic review of cases published between 1997 and 2017 identified only 23 instances of spontaneous regression; of these, nearly half involved optic pathway gliomas, and regression times ranged from three months to over 15 years [10].

Documented cases outside these contexts are virtually absent; only one adult supratentorial LGG with spontaneous regression has been reported in the literature [4].

Our case involves a 21-year-old woman with near-complete radiological regression of a supratentorial LGG without adjuvant therapy. This, together with the prior adult case, challenges current understanding of glioma behavior and underscores the need to explore underlying mechanisms and clinical management implications.

Mechanisms of spontaneous regression

Multiple hypotheses have been proposed to explain spontaneous glioma regression, particularly in pediatric and NF1-associated tumors:

Biopsy-induced immune activation: Surgical manipulation may provoke localized immune responses. Pediatric brainstem gliomas have shown regression post-biopsy, suggesting immune-mediated control [11-14].

Steroid or antiepileptic effects: While corticosteroids reduce edema, their brief use in this patient makes them an unlikely driver of lasting regression. Lamotrigine has no documented antitumoral properties, further diminishing this hypothesis [15].

Intrinsic apoptosis and ischemia: Observations in cerebellar astrocytomas link apoptotic markers and ischemic changes to tumor involution. Our histological findings, low Ki-67 (<1%) and focal lymphocytic infiltrates, support intrinsic apoptotic activity enhanced by immune mechanisms [7,8,16].

Cannabinoid effects: Preclinical studies show that Δ⁹-tetrahydrocannabinol (THC) and cannabidiol (CBD) can induce apoptosis, autophagy, and angiogenesis inhibition in glioma cells, often with synergistic effects in vivo [7,16-20].

However, in our case, cannabis exposure was limited to a sibling (smoking more than five times daily), with no patient use, making a direct therapeutic role unlikely.

Clinical implications and management

In clinical practice, the rarity of spontaneous LGG regression alone does not justify a passive “wait and see” strategy. Instead, current guidelines recommend a structured, personalized surveillance approach.

MRI every three months for the first two years, then every six to 12 months up to year five, and annually or every one to two years thereafter, following the National Institute for Health and Care Excellence (NICE) guideline for Grade II gliomas [21].

Surveillance should be tailored based on molecular risk factors defined in the 2021 WHO classification (IDH mutation, 1p/19q co-deletion, TERT/EGFR alterations), which inform prognosis and imaging intervals.

Advanced imaging (MR spectroscopy, perfusion MRI, PET) can be used when conventional MRI is inconclusive. Importantly, frequent imaging can increase patient anxiety, so shared decision‑making and psychological support are crucial [22].

Finally, while observational monitoring may be feasible in select young patients with favorable molecular and clinical features, evidence such as from a Norwegian cohort suggests that early resection offers superior overall survival [23], warranting prompt intervention if changes are detected. Thus, an individual-focused surveillance protocol that incorporates molecular profiling and patient preferences enables cautious optimism while maintaining readiness for timely treatment.

Limitations and future directions

A key limitation of these reports is the lack of molecular analysis. Future studies should explore predictive biomarkers of spontaneous regression, which could significantly influence therapeutic strategies.

## Conclusions

This case illustrates a rare instance of radiological regression in an adult with a diffuse low-grade glioma, observed in the absence of any adjuvant therapy. While this phenomenon suggests the possibility of spontaneous regression, such interpretation should remain cautious given the limitations inherent to single observations and the lack of histological confirmation of regression. Further long-term clinical and radiological follow-up, as well as the accumulation of similar documented cases, are necessary to better understand the biological mechanisms underlying this unusual evolution and to determine its clinical significance.

## References

[1] Duffau H, Taillandier L (2015). New concepts in the management of diffuse low-grade glioma: proposal of a multistage and individualized therapeutic approach. Neuro-Oncology.

[2] Gue R, Lakhani DA (2024). The 2021 WHO classification of tumours of the central nervous system. Biomedicines.

[3] Ishihara M, Yamamoto K, Miwa H, Nishi M (2017). Spontaneous complete regression of a brain stem glioma pathologically diagnosed as a high-grade glioma. Childs Nerv Syst.

[4] Scheer M, Spindler K, Emmer A (2022). Spontaneous remission of a “diffuse glioma”: a case report. Interdiscip Neurosurg.

[5] Gunny RS, Hayward RD, Phipps KP, Harding BN, Saunders DE (2005). Spontaneous regression of residual low-grade cerebellar pilocytic astrocytomas in children. Pediatr Radiol.

[6] Rozen WM, Joseph S, Lo PA (2008). Spontaneous regression of low-grade gliomas in pediatric patients without neurofibromatosis. Pediatr Neurosurg.

[7] Andradas C, Truong A, Byrne J, Endersby R (2021). The role of cannabinoids as anticancer agents in pediatric oncology. Cancers (Basel).

[8] Altinoz MA, Ozpinar A, Elmaci I (2019). Reproductive epidemiology of glial tumors may reveal novel treatments: high-dose progestins or progesterone antagonists as endocrino-immune modifiers against glioma. Neurosurg Rev.

[9] Piccirilli M, Lenzi J, Delfinis C, Trasimeni G, Salvati M, Raco A (2006). Spontaneous regression of optic pathways gliomas in three patients with neurofibromatosis type I and critical review of the literature. Childs Nerv Syst.

[10] Ahmed SI, Bareeqa SB, Samar SS, Jilanee SDA (2020). Resolving mystery behind autonomous retrogression of low-grade gliomas: a systematic review. J Pak Med Assoc.

[11] Samadian M, Bakhtevari MH, Haddadian K, Alavi HA, Rezaei O (2016). Spontaneous complete regression of hypothalamic pilocytic astrocytoma after partial resection in a child, complicated with Stevens-Johnson syndrome: a case report and literature review. Neurosurg Rev.

[12] Colosimo C, Cerase A, Maira G (2000). Regression after biopsy of a pilocytic opticochiasmatic astrocytoma in a young adult without neurofibromatosis. Neuroradiology.

[13] Kernan JC, Horgan MA, Piatt JH, D'Agostino A (1998). Spontaneous involution of a diencephalic astrocytoma. Pediatr Neurosurg.

[14] Lenard HG, Engelbrecht V, Janssen G, Wechsler W, Tautz C (1998). Complete remission of a diffuse pontine glioma. Neuropediatrics.

[15] Peddi P, Ajit NE, Burton GV, El-Osta H (2016). Regression of a glioblastoma multiforme: spontaneous versus a potential antineoplastic effect of dexamethasone and levetiracetam. BMJ Case Rep.

[16] Foroughi M, Hendson G, Sargent MA, Steinbok P (2011). Spontaneous regression of septum pellucidum/forniceal pilocytic astrocytomas--possible role of Cannabis inhalation. Childs Nerv Syst.

[17] Dorris K, Channell J, Hemenway M (2018). QOL-52. Use of cannabinoids in the pediatric central nervous system tumor population. Neuro-Oncology.

[18] Sredni ST, Huang CC, Suzuki M, Pundy T, Chou P, Tomita T (2016). Spontaneous involution of pediatric low-grade gliomas: high expression of cannabinoid receptor 1 (CNR1) at the time of diagnosis may indicate involvement of the endocannabinoid system. Childs Nerv Syst.

[19] Soto-Mercado V, Mendivil-Perez M, Jimenez-Del-Rio M, Fox JE, Velez-Pardo C (2020). Cannabinoid CP55940 selectively induces apoptosis in Jurkat cells and in ex vivo T-cell acute lymphoblastic leukemia through H(2)O(2) signaling mechanism. Leuk Res.

[20] Torres S, Lorente M, Rodríguez-Fornés F (2011). A combined preclinical therapy of cannabinoids and temozolomide against glioma. Mol Cancer Ther.

[21] National Institute for Health and Care Excellence (NICE) (2018). Brain Tumours (Primary) and Brain Metastases in Over 16s: Diagnosis and Management. https://www.nice.org.uk/guidance/ng99/chapter/Recommendations.

[22] Saha D, Nath AL, Trivedi AS (2025). Outcome survival following surgical resection of low-grade glioma. SSR Inst Int J Life Sci.

[23] Jakola AS, Myrmel KS, Kloster R, Torp SH, Lindal S, Unsgård G, Solheim O (2012). Comparison of a strategy favoring early surgical resection vs a strategy favoring watchful waiting in low-grade gliomas. JAMA.

